# Study protocol: SWING – social capital and well-being in neighborhoods in Ghent

**DOI:** 10.1186/s12939-015-0163-1

**Published:** 2015-04-09

**Authors:** Wim Hardyns, Veerle Vyncke, Lieven Pauwels, Sara Willems

**Affiliations:** Department of Criminology, Criminal Law and Social Law, Ghent University, Universiteitstraat 4 – 9000, Gent, Belgium; Research Foundation Flanders, Egmontstraat 5 - 1000, Brussels, Belgium; Department of Family Medicine and Primary Health Care, Ghent University, UZ- 6 K3 - De Pintelaan 185 – 9000, Gent, Belgium; Department of Criminology, Free University of Brussels, Pleinlaan 2 – 1050, Brussel, Belgium; Faculty of Law, Antwerp University, Venusstraat 23 – 2000, Antwerpen, Belgium

**Keywords:** Social capital, Neighborhood, Health, Inequity, Well-being

## Abstract

**Background:**

Investing in social capital has been put forth as a potential lever for policy action to tackle health inequity. Notwithstanding, empirical evidence that supports social capital’s role in the existence of health inequity is limited and inconclusive. Furthermore, social capital literature experiences important challenges with regard to (1) the level on which social capital is measured and analyzed; (2) the measurement of the concept in line with its multidimensional nature; and (3) the cross-cultural validity of social capital measurements.

The Social capital and Well-being In Neighborhoods in Ghent (SWING) study is designed to meet these challenges. The collected data can be used to investigate the distribution of health problems and the association between social capital, health and well-being, both at the individual and at the neighborhood level. The main goals of the SWING study are (1) to develop a coherent multilevel dataset of indicators on individual and neighborhood social capital and well-being that contains independent indicators of neighborhood social capital at a low level of aggregation and (2) to measure social capital as a multidimensional concept. The current article describes the background and design of the SWING study.

**Methods/Design:**

The SWING study started in 2011 and data were collected in three cross-sectional waves: the first in 2011, the second in 2012, and the third in 2013. Data collection took place in 142 neighborhoods (census tract level) in the city of Ghent (Flanders, Belgium). Multiple methods of data collection were used within each wave, including: (1) a standardized questionnaire, largely administered face-to-face interviews for neighborhood inhabitants (N = 2,730); (2) face-to-face interviews with key informants using a standardized questionnaire (N = 2,531); and (3) an observation checklist completed by the interviewers (N = 2,730 in total). The gathered data are complemented by data available within administrative data services.

**Discussion:**

The opportunities and ambitions of the SWING study are discussed, together with the limitations of the database.

**Electronic supplementary material:**

The online version of this article (doi:10.1186/s12939-015-0163-1) contains supplementary material, which is available to authorized users.

## Background

Social epidemiologists consistently find a relationship between socio-economic factors and health: the odds of ill health and premature death increase with a declining social position [1-7]. This association is more than the existence of a health gap between those worse and those better off, but typically follows a stepwise course [8,9]. The systematic health differences between socio-economic groups, which are socially produced and unfair, are referred to with the term ‘health inequity’ [9].

Recent research claims that health inequity is associated with substantial economic costs (estimated at a yearly cost of 9.4 % of the GDP), due to increased costs in healthcare, social security, and reduced labor productivity [10]. Tackling health inequity might be an interesting political strategy to meet the current economic crisis [11] and is one of the goals of the recent policy framework in het WHO European Region, Health 2020 [12]. An investment in social capital has been put forth as a potential lever for policy action [12,13]. Notwithstanding, empirical evidence that supports social capital’s role in the existence of health inequity is limited and inconclusive [14].

This methodological article describes the background and design of the Social capital and Well-being In Neighborhoods in Ghent (SWING) study, a cross-sectional study intended to collect detailed information on social capital at the individual and neighborhood level. The data of the SWING study will be used to explore social capital’s association with health and well-being and it’s impact on health inequity.

### Social capital, health and well-being

The term ‘social capital’ is used to refer to a number of social characteristics that involve the resources embedded in social networks [15]. It refers to the idea that social networks are a potential resource for individuals, communities, and the society as a whole [16].

At both the individual and collective level, social capital has been related to different aspects of health and well-being [17-21]. With respect to physical health, the strongest (positive) association is found between components of social capital and self-rated health. Evidence with regard to all cause mortality, morbidity, life expectancy, health behavior and mental health outcomes is less strong [22-24]. Most studies find a positive association between social capital on the one hand and health and well-being, although social capital’s health damaging influence has also been acknowledged [25-27].

Literature suggests that the flipside of social capital, i.e. its negative association with health outcomes, is more likely to occur in homogeneous and closed networks, due to the presence of health damaging social norms, higher levels of norm conformity and restricting levels of social control [28-30]. Different ‘types’ of social capital, i.e. bonding and bridging social capital, have been distinguished, based on the level of tie strength and levels of homogeneity of the social networks that lie at the basis of social capital. While bonding social capital is generally used to refer to social capital within homogeneous and closed social networks, bridging social capital refers to social capital between individuals that are linked through heterophilious and more open ties [31,32]. The latter provides access to non-redundant information and resources, while the first is believed to be more efficient in the provision of social support [32,33]. Since homogeneous ties are more easily formed [34], building bridging social capital probably requires additional attention. However, the balancing of bonding, bridging and linking social capital cannot be separated from the wider social climate in which individuals are embedded. For instance, high levels of income inequality are considered to be detrimental for the formation of social ties between the “haves” and “have-nots” (i.e. levels of bridging and linking social capital) since they lead to more pronounced social divides and experiences of unfairness [35-37].

### Social capital and health inequity

A detailed insight in the factors that explain the consistent link between socio-economic factors and health is needed to develop and implement effective strategies to tackle health inequities [38]. There is a general consensus that health inequities can mainly be explained by the joint effect of material conditions, individual health behavior and psychosocial factors [39-42]. Material conditions refer to characteristics of the physical environment, such as working and housing conditions, as well as factors associated with economic hardship. The next set of factors refer to individual health behaviors, including smoking, diet, physical activity and alcohol use. The psychosocial factors refer to diverse aspects such as social support, perceptions of social exclusion and the presence of psychosocial stressors such as low levels of job control [43].

The influence of social capital has mainly been mentioned in the context of the psychosocial indicators of health inequity. Neighborhood socioeconomic disadvantage has been associated to morbidity and mortality via declining levels of social cohesion and informal social control [44], which can both be considered as components of social capital. Furthermore, social capital has been identified as a stress-buffering concept, which might be particularly influential for the health of people living in deprived circumstances [45-47]. However, levels of social capital are also believed to influence the two other main pathways that contribute to health inequity, being health behavior and material factors. Literature shows that social capital influences health behavior trough the values and norms with regard to health within social networks [23,48,49]. Furthermore, higher levels of social capital have been associated with upwards social mobility (e.g. via the spread of information on job openings) and can provide access to different material resources that people do not possess themselves [28,50].

### The SWING study: rationale and research aims

The study of social capital in explaining health and well-being has moved to front stage in political debates and in the context of scientific evidence [51]. However, some challenges can be pointed out regarding (1) the level on which social capital is measured and analyzed in studies; (2) the measurement of the concept in line with it’s multidimensional nature; and (3) the cross-cultural validity of social capital measurements [52,53].

The SWING study has been designed in an attempt to address these gaps in current literature. The general objective of the SWING study is twofold:

1. To develop a coherent multilevel dataset of indicators on individual and neighborhood social capital and health and well-being that contains independent indicators of neighborhood social capital at a low level of aggregation;
2. To measure social capital in line with the multidimensional nature of the concept.

In this paragraph, we further discuss the main challenges for social capital research. First, there is no consensus in literature concerning the operational level on which social capital is measured and analyzed [32,33,51,54,55]. While some scholars define social capital as an attribute of individuals, others consider it as a collective attribute (i.e. measured at the level of neighborhoods, communities or even entire societies) [51,56]. Despite theoretical and empirical support for both the individual and collective dimension of social capital, individual-based theories have largely ignored community-level influences, while community-based theories have belittled the importance of individual influences [57]. This theoretical bifurcation is considered unfruitful [51]: social capital is likely to influence health and well-being at the both levels. In addition, research has revealed complex cross-level interactions between social capital measured at the individual and collective level [56,58]. Scholars increasingly admit the need to study social capital within a multilevel framework to gain a more detailed insight into the exact role social capital plays in the distribution of health and well-being [51].

Furthermore, the geographical level on which collective social capital is measured might be improved upon. Scholars are often forced to operationalize social capital at rather large levels of analysis, such as the national level or the city level, for the simple reason that statistical material tends to be merely available at this level. However, this hinders an exploration of small-area differences in the distribution of health and well-being [44,59,60]. In that sense, it is praiseworthy that more recent studies on social capital are extensively using neighborhoods (or census tracts) as units of analysis. However, more granular studies that use even smaller units of analysis, for example street-blocks, are still very scarce in the social capital domain.

Another point of interest concerning the unit of analysis refers to the aggregation method of measuring neighborhood social capital. In most studies, the community-level measures of social capital are simply the aggregates of the individual-level measures (using mean scores). However, independent measurement methods for neighborhood social capital should be preferred [61-63]. The SWING study gathers data on social capital and health and well-being within a multilevel framework. Furthermore, the key informant technique (see further) is used to gather objective measures of social capital at the level of local neighborhoods.

Secondly, there is a need to broaden the scope of social capital measurements. Different views on the core element of social capital can be distinguished in literature, which can be attributed to the interdisciplinary background of researchers who study the concept. Most empirical studies on social capital and health and well-being consider social norms within networks, such as trust and reciprocity, as the core of social capital [51,64]. However, this focus has been subject to critique since it easily ignores the potential downside of social capital for health and well-being and the influence of social stratification on the access to and use of social capital [26,65]. Consequently, some researchers have proposed a shift in social capital theory from a ‘normative’ to a ‘resource-based’ perspective [66-68]. The latter identifies the resources embedded in social networks as the core of the concept [67] and has some important benefits over the ‘normative’ approach. Due to its strict focus on resources in social networks, this vision enables a clear distinction of social capital from its antecedents and consequences, and facilitates the elaboration of testable hypotheses on social capital and health and well-being [65,66]. The ‘resource-based’ approach to social capital is considered especially useful to study social capital’s role for health inequity since it incorporates the influence of social position on the access to and use of social capital [26,67]. The SWING study contains a multidimensional set of indicators of social capital, which fit both within the normative and resource-based approach to social capital.

Finally, empirical research on social capital and health and well-being mostly stems from Canada, the USA or Scandinavian countries. However, differences in the cultural and political climate between countries might affect the influence social capital has on health and well-being [69,70]. For instance, the relationship between components of social capital and health is known to depend upon welfare state type [28]. Theories on social capital cannot implicitly be transferred from one context to another [71,72] and evidence from contexts comparable to Belgium is very scarce. For that reason, the questionnaires of the SWING study are as much as possible adapted to the context of this study, which is a Western-European medium-sized city.

### Methods/design

The data for this study were collected in the city of Ghent, in three successive cross-sectional waves of data collection: SWING 1 in 2011, SWING 2 in 2012 and SWING 3 in 2013. Although the content of the questionnaires used in the SWING study was partially identical in all three data collection waves, each separate data collection wave had a slightly different focus with regard to included outcome and control variables. We come back to this when we discuss the questionnaire development.

Multiple methods of data collection were used within each wave:

1. At the individual level of inhabitants, data were collected by means of *face-to-face interviews*. Data were largely collected using structured questionnaires that were administered face-to-face. Additionally, respondents were presented some possibly sensitive questions (e.g. questions about income and substance use) in a short self-administered questionnaire. This method was used for the measurement of individual social capital, and health and well-being outcomes.
2. At the neighborhood level, data were gathered using the *key informant technique* through a face-to-face standardized questionnaire. This method was used for the measurement of neighborhood social capital, and other neighourhood processes of social (dis)organization. Furthermore, an *observation checklist* was completed by the interviewers to evaluate the facilities and (green) space in the neighborhood.
3. The collected data were complemented by (mainly) *administrativedata* from existing, external databases from the City of Ghent and Ghent University. These data were gathered to have information on the social and economic structure of the neighborhood.

The fieldwork of this study was conducted by trained interviewers within the framework of their methodology classes (2nd bachelor criminological sciences). After an intensive interview training and teaching of the survey methodology, the students were divided in groups. Each group was responsible to collect data in one specific neighborhood. The interview with the neighborhood inhabitants and the observation checklist were completed during a home visit. After each interview, the characteristics of the living environment of the respondent (e.g., green and recreational facilities, disorder, etc.) were evaluated using a standardized observation checklist. The interviewers were asked to fill in the checklist in the absence of the respondents. The interviews with key informants were performed at the most convenient location for the participants, which was generally their work place. In total, 504 interviewers contributed to this study: 164 interviewers in 2011; 161 interviewers in 2012; and 179 interviewers in 2013 [See Additional file 1 for more information about the data collection procedure]. In 2014, extra interviews with key informants (N = 1,131) were executed by 142 interviewers in the same neighborhoods. We found high correlational validities of the measurements from different waves of data collection, which suggest that we actually measured what we wanted to measure.

### Setting

Ghent is a densely populated city in the northern part of Belgium. It is the second-largest municipality in Belgium, and it covers 158 km^2^ with a population of approximately 250,000 residents. The municipality is divided into 201 statistical sectors, from which 142 statistical sectors have a minimum population size of 200 adult inhabitants. To operationalize neighborhoods, the current study used statistical sectors, which are comparable to the census tract level in the US or the UK and the smallest administrative unit of analysis on which objective administrative data (demographic, social, and economic indicators) are available (infra the term “neighborhood” will be used instead of statistical sector).

### Sampling of neighborhoods

**Figure 1:**
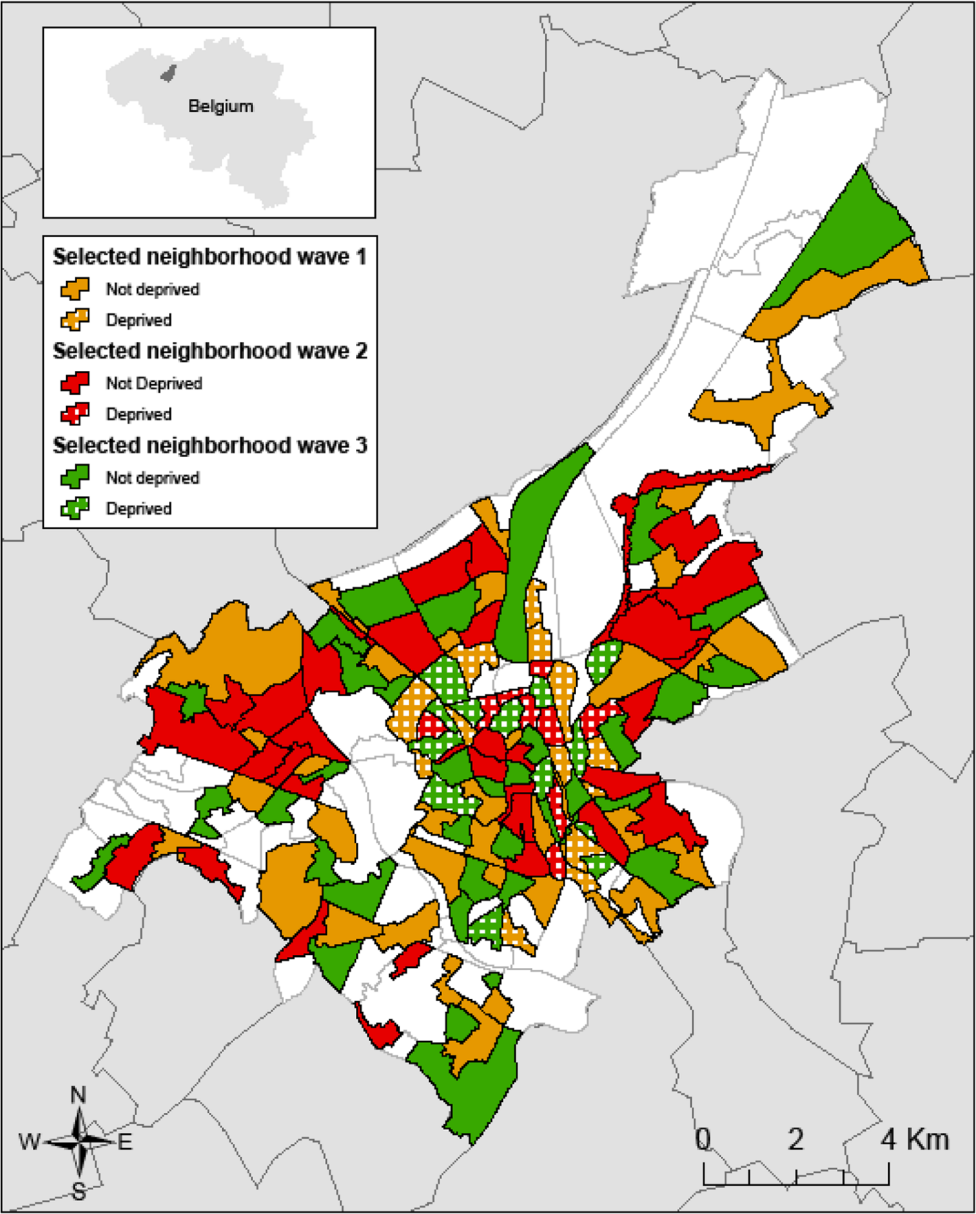
**Geographical distribution of the selected neighborhoods.**

In each year of data collection a stratified sample of 50, 42 and 50 neighborhoods respectively was selected from the 142 neighborhoods in Ghent with a minimum population size of 200 adult inhabitants [see Figure 1]. Each neighborhood could only be included in one of the three data collection waves of the SWING study. Neighborhoods were selected following a stratified selection procedure based on population density and the level of deprivation (deprived versus non-deprived), resulting in a representative set of neighborhoods for each sample [see Table 1]. Information on deprivation level was based on information from tax and census databases and takes socio-economic data, the population composition, and the physical characteristics of the neighborhood into account [73] [see Additional file 2]. For each data collection wave, spatial proximity was minimized as much as possible. When bordering neighborhoods had to be selected because there are no isolated, unselected neighborhoods left, preference was given to neighborhoods which are separated by clear geographical boundaries, such as major roads or bridges.

**Table 1:** **Criteria for the selection of neighborhoods**

***Population density (inhabitants/km*** ^***2***^ ***)***	***N neighborhoods with minimum population size of 200 inhabitants (N deprived neighborhoods)***	***N selected neighborhoods 2011 (N deprived neighborhoods)***	***N selected neighborhoods 2012 (N deprived neighborhoods)***	***N selected neighborhoods 2013 (N deprived neighborhoods)***
*≤1000*	27 (0)	9 (0)	9 (0)	9 (0)
*1000-1999*	21 (1)	7 (1)	7 (0)	7 (0)
*2000-2999*	18 (0)	6 (0)	6 (0)	6 (0)
*3000-3999*	13 (4)	5 (1)	3 (1)	5 (2)
*4000-4999*	13 (3)	5 (1)	3 (1)	5 (1)
*≥5000*	50 (27)	18 (10)	14 (7)	18 (10)
***Total***	***142 (35)***	***50 (13)***	***42 (9)***	***50 (13)***

### Sampling of inhabitants

The inclusion criteria to participate as a neighborhood inhabitant in this study were: (1) being older than 18; (2) not living in an institutional setting (e.g., a home for the elderly, prison, etc.); and (3) having sufficient knowledge of the Dutch language to complete the questionnaire. Except for the final criterion, this information was derived from the municipal registry and taken into consideration in the sampling. Language proficiency was determined at the moment of first contact.

For the *face-to-face interviews* with inhabitants, a randomized sample was drawn from the municipal registry for each of the selected neighborhoods. This sample was representative of the composition of each neighborhood, stratified by age (18–24, 25–34, 35–44, 45–54, 55–64, 64–74, 75+), sex (male versus female), and current nationality (Belgian versus non-Belgian). For each selected inhabitant, three substitutes were selected within the same category with regard to age, sex, and nationality. Respondents who couldn’t be reached after three home visits or refused to participate were replaced by a randomly selected respondent from the corresponding age, gender, and ethnic stratum.

The ambition was to gain the participation of 20 inhabitants in each of the 142 neighborhoods. In 2011, 1,025 neighborhood inhabitants from 50 neighborhoods were interviewed; in 2012, 762 neighborhood inhabitants from 42 neighborhoods were interviewed; and in 2013, 943 neighborhood inhabitants from 50 neighborhoods were interviewed [see Tables 2 and 3]. In total, 2,730 neighborhood inhabitants from 142 neighborhoods were reached. The overall response rate is 47,89%.

**Table 2:** **Percentage of neighborhood inhabitants reached in each data collection wave**

	**% wave 1 (2011)**	**% wave 2 (2012)**	% wave 3 (2013)*
**Original sample**	49	47	40
**1st substitutes sample**	27	26	22
**2nd substitutes sample**	15	14	13
**3rd substitutes sample**	8	10	10
**More than 3 substitutes**	1	3	15
**Total**	100	100	100

*In wave 3 (2013) five substitutes samples were at the disposal of the interviewers, whereas there were only three substitutes samples in wave 1 (2011) and wave 2 (2012).

**Table 3:** **Overview of neighborhood inhabitants’ characteristics**

	**N 2011**	**% 2011**	**N 2012**	**% 2012**	**N 2013**	**% 2013**
**Sex**						
Male	494	48.2	370	48.6	446	47.3
Female	530	51.8	392	51.4	497	52.7
**Nationality**						
Belgian	915	89.3	703	92.3	846	89.8
Non-Belgian	110	10.7	59	7.7	96	10.2
**Educational level**						
Low	198	19.4	130	17.1	182	19.7
Middle	378	37.0	263	34.7	271	29.3
High	445	43.6	366	48.2	472	51.0
**Paid work**						
Yes	603	59.4	454	59.6	556	59.0
No	412	40.6	308	40.4	386	41.0
**Age**						
18-24	100	9.8	82	10.8	90	9.5
25-34	213	20.9	135	17.7	199	21.1
35-44	185	18.1	124	16.3	161	17.1
45-54	179	17.5	124	16.3	129	13.7
55-64	136	13.3	116	15.2	135	14.3
65-74	102	10.0	93	12.2	116	12.3
75+	105	10.3	87	11.4	113	12.0

### Sampling of key informants

At the neighborhood level, data were gathered using the *key informant technique*. This technique has the potential to create ecologically reliable and valid measures of neighborhood social processes [61] which are not simply the aggregate of individual-level measures. Key informants are defined as “persons who are in a ‘privileged’ position to provide detailed information on local area processes” (p. 404) [61] and can be described as privileged witnesses. They often have more knowledge about the social processes under consideration than the average inhabitant, and provide more useful and less biased information. Examples of good key informants are family doctors, police officers on the beat, local community and postal workers, managers of local shops, café or pub owners, and staff of other local catering industries. In contrast to the sample of neighborhood inhabitants, who were selected by random stratified sampling, the key informants were purposely chosen by the interviewers on the basis of their supposed knowledge about the social processes studied in the neighborhood. The selection of good key informants was a topic that was covered during the interviewer training. Each interviewer was provided with a detailed non-limitative overview of possible key informants, but were encouraged to select other key informants with supposedly good knowledge on neighborhood processes. They were encouraged to contact the research team in case of doubt about the eligibility of key informants. Because key informants generally have an above average knowledge of the social processes under study compared to neighborhood inhabitants, fewer key informants are needed to create ecologically sound measures [61].

In this study, we strove for a heterogeneous set of 8 to 10 key informants per neighborhood. To be included in this study, key informants had to meet the following inclusion criteria: (1) being older than 18; (2) having sufficient knowledge of the Dutch language to complete the questionnaire; and (3) being in a work position that presumes an above average knowledge of the social processes in one of the neighborhoods studied. In total, 638 key informants were included in 2011; 360 key informants were included in 2012; and 402 key informants were included in 2013 [see Table 4]. In 2014, another 1,131 key informants were interviewed to validate the results from the previous data collections. In total, 2,531 key informants from 142 neighborhoods were reached.

**Table 4:** **Overview of key informants’ characteristics**

	**N 2011**	**% 2011**	**N 2012**	**% 2012**	**N 2013**	**% 2013**
**Sex**						
Male	268	42.0	141	39.3	179	44.5
Female	370	58.0	218	60.7	223	55.5
**Age**						
18-24	46	7.3	13	3.6	29	7.2
25-34	129	20.3	76	21.1	80	19.9
35-44	150	23.7	89	24.7	104	25.9
45-54	205	32.3	102	28.3	108	26.9
55-64	82	12.9	69	19.2	60	14.9
65-74	19	3.0	10	2.8	19	4.7
75+	3	0.5	1	0.3	2	0.5
**Length of activity in neighborhood**						
<1 year	69	10.9	30	8.3	30	7.5
>1 year & < 5 years	150	23.6	80	22.2	114	28.4
>5 years & < 10 years	118	18.6	65	18.1	73	18.2
>10 years	298	46.9	185	51.4	185	46.0

### Questionnaire development

Although the content of the questionnaires used in the SWING study was partially identical in all three data collection waves, each separate data collection wave had a slightly different focus with regard to included outcome and control variables. For example, the 2011 questionnaire contained measurements of personality characteristics (extraversion, agreeableness, conscientiousness, neuroticism and openness), locus of control, use of psychopharmaca, financial barriers to health care and procedural justice. On the other hand, the 2012 questionnaire focused more on medical resources and police satisfaction, while the 2013 questionnaire contained information of multimorbidity, criminal victimization and aspects of transport and transit. This enabled us to study the impact of social capital on a broad of aspects of health and well-being and vice versa. The sampling procedure was performed in such a manner that one can either analyze the data of each data collection wave separately, or that the different data collection waves can be merged to one overarching database. The general overlapping questionnaire of neighborhood inhabitants and key informants, i.e. the questions that were included in all three data collection waves, can be found in Additional file 3 and Additional file 4 respectively. The specific questionnaires for the different data collection waves can be received upon request.

The questionnaires for neighborhood inhabitants and key informants were largely based on existing and validated questionnaires on social processes, both nationally and internationally, such as the Resource Generator [74], the Social Capital Community Benchmark Survey (The Saguaro Seminar of Robert Putnam) [75], the MOS Social Support Survey [76], the European Social Survey [77], the Project on Human Development in Chicago Neighborhoods Community Survey [78], the Survey on the Social Networks of the Dutch [79], the Belgian Security Monitor, and the Social Cohesion Indicators in Flanders Survey [80]. The observation checklist was based on the questionnaire that was used in the research project Vitamin G, which studies the local green facilities in urban neighborhoods and their relation with health, well-being, and social safety [81], and can be found in Additional file 3. This observation checklist has proven to be of major importance in generating reliable indicators of green areas and streetscape greenery in neighborhood studies [81,82].

Because some of the instruments used were not available in Dutch, a standard translation procedure was set up. Before the questionnaire for neighborhood inhabitants was completed, cognitive interviews were used as a method of instrument testing. This led to minor changes to the questionnaire – mainly to specific terms and expressions, as well as layout – before further use in the study [See Additional file 5 for more information about the translation procedure of the questionnaires and the cognitive interviews].

### External data

Additional *administrative data* concerning the neighborhoods were joined with the neighborhood data from the key informants and the observation checklist. These external data were made available by administrative agencies and comprise a multitude of social and structural indicators: demographic (gender, age, household size, residential mobility/turnover), structural (residential density, percentage of green zones), socio-economic (ethnic minorities, mean income, income inequality, unemployment) and other (crime statistics, walkability) indicators.

### Data processing

All information regarding data handling and data construction can be found in Additional file 6.

## Discussion

The SWING study will inform both researchers and policy makers on the relationship between social capital and indicators of well-being such as mental health, self-perceived health, health risk behaviors, and avoidance behavior. The data can be used to explore social capital’s general association with health and well-being, social capital’s role for health inequity and the determinants that influence individual and neighborhood stocks of social capital.

It is the ambition of the SWING study to answer to the gaps in current literature by developing a large and coherent multidimensional and detailed dataset of social capital, which contains data both at the individual and at the neighborhood level. An important strength of the current study is the collection of data at a small level of analysis, which enables a detailed study of small area differences in health, well-being and social capital.

Although the design of the SWING study overcomes some limitations in present social capital literature, some weaknesses should also be considered. The data from the SWING study are cross-sectional in nature. The data will not enable the analysis of trends in time or the unraveling of causal relationships. Furthermore, the population in the study is quite specific, since the data are gathered in one medium-sized Western-European city. And at last, it is likely that our data underestimate the multicultural composition of the population. Additional non-response analyses show that respondents with the Belgian nationality are slightly overrepresented in our final sample, which suggests a relatively higher likelihood to be excluded from the sample for people with a non-Belgian nationality. The fact that respondents had to have a basic proficiency of the Dutch language might explain this observation. As such, the data from the SWING study are not the most suited to explore the differential association between social capital and health for different ethnic subpopulations. A focused study on social capital and health among ethnic minorities in Ghent could be useful to complement the data from the SWING study. If possible, future research should try to safeguard representativeness of their study sample with regard to ethnicity, e.g. by providing questionnaires in different languages. Despite these limitations, however, the SWING study is believed to contribute to an understanding of the association between individual and neighborhood social capital on the one hand and well-being on the other.

## Additional files

Additional file 1:
**Background data collection procedure.**
Additional file 2:
**Sampling of neighborhoods.**
Additional file 3:
**Neighborhood inhabitants questionnaire and observation checklist.**
Additional file 4:
**Key informant questionnaire.**
Additional file 5:
**Questionnaire construction.**
Additional file 6:
**Data processing.**
