# Supplementary material for: Study protocol: SWING – social capital and well-being in neighborhoods in Ghent
Source: Int J Equity Health. 2015 Apr 9;14:36. doi: 10.1186/s12939-015-0163-1 (PMC4437247; doi:10.1186/s12939-015-0163-1)
Supplement: Additional file 1: — Background data collection procedure. [file 12939_2015_163_MOESM1_ESM.docx]

**Additional file 1: Background data collection procedure**

First, an information letter containing background information and the rationale for the study was sent to all selected neighborhood inhabitants by mail. This letter also announced that an interviewer wearing an identification badge would visit them at home. In the following weeks, each respondent was visited at home by an interviewer and invited to participate. Respondents who were willing to participate were asked to complete the survey. The questionnaire was partly administered face-to-face. Questions that were too sensitive and would likely lead to higher non-response during a face-to-face administration (e.g., questions on income and financial difficulties, alcohol- and drug-use) were gathered in a short self-administered questionnaire that was handed over to the respondents after completion of the face-to-face part.

The key informants were personally invited to participate by the interviewers, by email, by phone, or face-to-face. Each received a similar information letter during the first face-to-face contact with the interviewer. The key informant questionnaire was administered face-to-face.
