# Supplementary material for: Study protocol: SWING – social capital and well-being in neighborhoods in Ghent
Source: Int J Equity Health. 2015 Apr 9;14:36. doi: 10.1186/s12939-015-0163-1 (PMC4437247; doi:10.1186/s12939-015-0163-1)
Supplement: Additional file 2: — Sampling of neighborhoods. [file 12939_2015_163_MOESM2_ESM.docx]

**Additional file 2: Sampling of neighborhoods**

Information on the deprivation level of neighborhoods is based on the Atlas of Deprived Neighborhoods. This atlas contains for each statistical sector in Flanders and Brussels information on 22 variables originating from tax and census databases and describes the population composition (proportion of migrants, house-owners, single-parent families, educational level of the population, and number of unemployed and manual workers, …); the houses in the statistical sector (number of rooms in the house, quality of the house, and having central heating); and the quality of the physical environment (air pollution, noise, and garbage in the streets). Based on these variables seven indicators are build using principle component analysis. For each indicator a threshold defined by experts in the field is determined. Sectors which score under the threshold for at least 4 indicators, are considered as deprived (35 deprived statistical sectors in Ghent) [[1](#_ENREF_1)].

**References**

1. Vandermotten C, Marissal P, Van Hamme G, Kesteloot C, Slegers K, Vanden Broucke L, Ippersiel B, de Bethune S, Naiken R: **Dynamische analyse van de buurten in moeilijkheden in de Belgische stadsgewesten**. Brussel: Grootstedenbeleid - POD Maatschappelijke Integratie; 2006.
