# Supplementary material for: Study protocol: SWING – social capital and well-being in neighborhoods in Ghent
Source: Int J Equity Health. 2015 Apr 9;14:36. doi: 10.1186/s12939-015-0163-1 (PMC4437247; doi:10.1186/s12939-015-0163-1)
Supplement: Additional file 3: — Neighborhood inhabitants questionnaire and observation checklist. [file 12939_2015_163_MOESM3_ESM.docx]

| **Additional file 3: Neighborhood inhabitants questionnaire and observation checklist** |
| --- |

**Neighbourhood inhabitants questionnaire: orally administered**

SOCIAL NETWORKS

1. How many people from your personal network of friends, family and acquaintances, if any, can you discuss important personal matters with?

About …………………..

1. Are you an active member of any clubs/associations? If so, how many? If you are not an active member of any club or association, please write ‘0’.

…………………………… clubs/associations

1. On an average, about how many people do you have contact with in a typical weekday, including all those who you say hello, chat, talk or discuss matters with, whether you do it face-to-face, by telephone, by mail or on the internet and whether you personally know the person or not? Please give an estimate of this number.

About …………………..

1. People can sometimes count on people they know (family, friends or acquaintances) for assistance or support. How many people can you count on for the following forms of assistance?

*Note: this is* ***not*** *about people that you pay to help you or professionals, but* ***only*** *people from your personal network of family, friends or acquaintances.*

| How many people from your personal network of family, friends or acquaintances … | 0 | 1 | 2 | 3 | 4 | 5 | 6-10 persons | > 10 persons |
| --- | --- | --- | --- | --- | --- | --- | --- | --- |
| 4.1 … understand your problems? | □ | □ | □ | □ | □ | □ | □ | □ |
| 4.2 … would let you move into their house for a week if you temporarily could not stay at your house? | □ | □ | □ | □ | □ | □ | □ | □ |
| 4.3 … would encourage you to go to the doctor if you experience health problems? | □ | □ | □ | □ | □ | □ | □ | □ |
| 4.4 … make you feel good (e.g. make you feel you are useful or make you feel that they are glad to know you)? | □ | □ | □ | □ | □ | □ | □ | □ |

1. We would like to know how many people in your personal network work in the health sector.

*Note: this is again* ***not*** *about people that you pay to help you/professionals, but* ***only*** *people from your personal network of family, friends or acquaintances.*

| How many people from your personal network of family, friends or acquaintances … | 0 | 1 | 2 | 3 | 4 | 5 | 6-10 persons | > 10 persons |
| --- | --- | --- | --- | --- | --- | --- | --- | --- |
| 5.1 … are a medical doctor? | □ | □ | □ | □ | □ | □ | □ | □ |

HEALTH AND WELL-BEING

1. How would you rate your health status in general?

| □ Very good | □Good | □ Fair | □ Bad | □ Very bad |
| --- | --- | --- | --- | --- |

- 1. Have you ever smoked cigarettes, cigars and/or pipes, during a long period of time (i.e. minimum 1 year consecutively) and (almost) every day?

| □ Yes | □ No |
| --- | --- |

- 1. Do you currently smoke?

| □ Yes, daily | □ Yes, occasionally | □ No |
| --- | --- | --- |

FEELINGS OF UNSAFETY

1. Does it happens that …

|  | Never | Seldom | Sometimes | Often | Very often |
| --- | --- | --- | --- | --- | --- |
| V8_1 … you avoid certain areas in your neighbourhood because you think they are not safe? | □ | □ | □ | □ | □ |
| V8_2 … you avoid opening the door to strangers because you think it is not safe? | □ | □ | □ | □ | □ |
| V8_3 … you avoid leaving home after dark because you think it is not safe? | □ | □ | □ | □ | □ |

SOCIODEMOGRAPHIC CHARACTERISTICS

| V9_1 In which year were you born? | 19………… | |
| --- | --- | --- |
| V9_2 Did you have the Belgian nationality at birth? | □ Yes | □ No |
| V9_3 Did your father have the Belgian nationality at birth? | □ Yes | □ No  Another nationality, being  ………..……….. |
| V9_4 Did your mother have the Belgian nationality at birth? | □ Yes | □ No  Another nationality, being  ………..……….. |
| V9_5 Do you currently have a partner? Whether or not you and your partner live together is not important for this question. | □ Yes | □ No |
| V9_6 Are you currently involved in payed work?  [*ENQ: also choose ‘yes’ if the respondent works part-time, or if his/her professional activity is temporally interrupted due to parental leave, sick leave, or other reasons.*] | □ Yes | □ No |

1. How many people live in this house, NOT including yourself? Please include children who don’t live here fulltime (e.g. due to joint custody, living at campus, …)

V10_1 Number of children younger than 14 years of age □□

V10_2 Number of children aged 14 or older, but younger than 18 years of age □□

V10_3 Number of people aged 18 or older (yourself not included) □□

1. Is this a rented house or do you own the property?

□ This house is rented, via social renting

□ This house is rented, via private renting

□ This house is my/our/… property

| **Neighborhood inhabitants questionnaire: self administered partim** |
| --- |

SOCIODEMOGRAPHIC CHARACTERISTICS

1. What is the highest level of education that you completed?

|  |  |
| --- | --- |
| No education or primary education | □ |
| Lower level of secondary education | □ |
| Higher level of secondary education | □ |
| Higher education | □ |

1. The following questions are about the total net disposable income of all members of your household together. This includes wages, salaries, benefits, allowances etc. of all household members.

V13_1 Please situate the total net monthly income of your household, using the following response categories.

□ € 0-499 □ € 3000-3499

□ € 500-999 □ € 3500-3999

□ € 1000-1499 □ € 4000-4499

□ € 1500-1999 □ € 4500-4999

□ € 2000-2499 □ € 5000 -7499

□ € 2500-2999 □ € 7500 – 9999

□ € 10.000 or more

V13_2 How easy is it for your household to make ends meet using the available monthly income?

| Very hard | Hard | Easy nor hard | Easy | Very easy |
| --- | --- | --- | --- | --- |
| □ | □ | □ | □ | □ |

**Observation checklist (to be completed by the interviewer)**

1. Green space in the street

V14_1. How would you rate the maintenance of the green space in this street?

| Very bad | 1 | 2 | 3 | 4 | 5 | Very good | No green space |
| --- | --- | --- | --- | --- | --- | --- | --- |

V14_2. How would you rate the variation in green space in this street?

| Very monotone | 1 | 2 | 3 | 4 | 5 | Very varied | No green space |
| --- | --- | --- | --- | --- | --- | --- | --- |

V14_3. Overall, how would you rate the green space in this street?

| Very negative | 1 | 2 | 3 | 4 | 5 | Very positive | No green space |
| --- | --- | --- | --- | --- | --- | --- | --- |

1. Course of the interview

To what extent do you agree with the following statements concerning the course of the interview?

|  | Absolutely disagree | Disagree | Agree nor disagree | Agree | Absolutely agree |
| --- | --- | --- | --- | --- | --- |
| V15_1 I think the respondent has understood everything. | □ | □ | □ | □ | □ |
| V15_2 The administration of the questionnaire went smoothly. | □ | □ | □ | □ | □ |

**To be completed by the interviewer**

1. In which neighborhood does the respondent live?

……………………………………………………………………………

1. Sex of the respondent

□ Male □ Female
