# Supplementary material for: Study protocol: SWING – social capital and well-being in neighborhoods in Ghent
Source: Int J Equity Health. 2015 Apr 9;14:36. doi: 10.1186/s12939-015-0163-1 (PMC4437247; doi:10.1186/s12939-015-0163-1)
Supplement: Additional file 4: — Key informant questionnaire. [file 12939_2015_163_MOESM4_ESM.docx]

| **Additional file 4: Key informant questionnaire** |
| --- |

**Key informant questionnaire: orally administered**

1. ID-number key informant?

*Note: to be assessed by the interviewer.*

1. In which neighborhood do you work?

*Note: only 1 of 142 Ghent neighborhoods can be chosen.*

1. For how long do you work in this neighborhood?

□ less than 1 year

□ more than 1 year and less than 5 years

□ more than 5 years and less than 10 years

□ more than 10 years

1. How satisfied are you with the presence in this neighborhood of …

|  | Very satisfied | Satisfied | Nor satisfied, nor unsatisfied | Unsatisfied | Very unsatisfied |
| --- | --- | --- | --- | --- | --- |
| V4_1 … green? | □ | □ | □ | □ | □ |
| V4_2 … facilities for young people between 12 and 18 years (e.g. soccer pitch, place to hang around, etc.)? | □ | □ | □ | □ | □ |
| V4_3 … play areas for young children? | □ | □ | □ | □ | □ |
| V4_4 … public transport? | □ | □ | □ | □ | □ |

1. How strongly do you agree (on a five-point scale) that …

|  | Totally agree | Agree | Nor agree, nor disagree | Disagree | Totally disagree |
| --- | --- | --- | --- | --- | --- |
| V5_1 … people around here are willing to help their neighbours? | □ | □ | □ | □ | □ |
| V5_2 … this is a close-knit neighbourhood? | □ | □ | □ | □ | □ |
| V5_3 … people in this neighbourhood can be trusted? | □ | □ | □ | □ | □ |
| V5_4 … contacts between inhabitants in this neighbourhood are generally positive? | □ | □ | □ | □ | □ |

1. How likely (on a five-point scale) do you think the neighbors in this neighborhood could be counted on to intervene in various ways if …

*Note: calling to the police can be conceived as a way to intervene.*

|  | Very likely | Likely | Nor likely, nor unlikely | Unlikely | Very unlikely |
| --- | --- | --- | --- | --- | --- |
| V6_1 … children were skipping school and hanging out on a street corner? | □ | □ | □ | □ | □ |
| V6_2 … children were spray-painting graffiti on a local building? | □ | □ | □ | □ | □ |
| V6_3 … children were showing disrespect to an adult? | □ | □ | □ | □ | □ |
| V6_4 … a fight broke out in front of their house? | □ | □ | □ | □ | □ |
| V6_5 … children were making too much racket? | □ | □ | □ | □ | □ |
| V6_6 … children were using soft drugs (smoking weed, hasj, etc.)? | □ | □ | □ | □ | □ |

1. How often (on a five-point scale) have you observed each of the following occurrences in this neighborhood?

|  | Never | Seldom | Sometimes | Often | Very often |
| --- | --- | --- | --- | --- | --- |
| V7_1 Adolescents hanging around on street corners. | □ | □ | □ | □ | □ |
| V7_2 Groups of adolescents harassing persons to obtain money or goods. | □ | □ | □ | □ | □ |
| V7_3 Men drinking alcohol in public (e.g. on a bus stop or a supermarket in this neighborhood). | □ | □ | □ | □ | □ |
| V7_4 Persons selling drugs (hash, weed, etc.) on the streets. | □ | □ | □ | □ | □ |
| V7_5 People being threatened on the streets with weapons or knives. | □ | □ | □ | □ | □ |
| V7_6 Fights between adolescents on the streets. | □ | □ | □ | □ | □ |
| V7_7 Men urinating in public (e.g. against walls of houses, bus infrastructure, etc.). | □ | □ | □ | □ | □ |
| V7_8 Litter on the streets. | □ | □ | □ | □ | □ |
| V7_9 Exhaust gas. | □ | □ | □ | □ | □ |
| V7_10 Bad smell (other than exhaust gas). | □ | □ | □ | □ | □ |

1. Does it happens that …

|  | Never | Seldom | Sometimes | Often | Very often |
| --- | --- | --- | --- | --- | --- |
| V8_1 … you avoid certain areas in this neighbourhood because you think they are not safe? | □ | □ | □ | □ | □ |
| V8_2 … you avoid to walk around in this neighborhood after dark? | □ | □ | □ | □ | □ |

1. Sex of the key informant?

*Note: To be assessed by the interviewer.*

□ Male

□ Female

1. In which year were you born?

| 1 | 9 |  |  |
| --- | --- | --- | --- |
