# Supplementary material for: Study protocol: SWING – social capital and well-being in neighborhoods in Ghent
Source: Int J Equity Health. 2015 Apr 9;14:36. doi: 10.1186/s12939-015-0163-1 (PMC4437247; doi:10.1186/s12939-015-0163-1)
Supplement: Additional file 5: — Questionnaire construction. [file 12939_2015_163_MOESM5_ESM.docx]

**Additional file 5: Questionnaire construction**

***Translation procedure of the questionnaires***

Some of the instruments used were not available in Dutch. A back-translation and a forward-translation procedure was followed. The original English items were translated into Dutch by an independent interpreter. The translated items were then retranslated into English by a member of the research team. A third independent researcher compared the retranslated English items and the original English items, and finalized the Dutch translations, which were as closely related to the original items as possible.

***Cognitive interviews***

This method, developed in the 1980s by survey methodologists and psychologists, is intended to evaluate sources of response error [[1](#_ENREF_1)]. It aims to clarify how the questions are understood and how judgments about responses are made, and to identify and explore any problems caused by the questions in the survey [[2](#_ENREF_2)]. The technique is recommended as a means to improve the validity of obtained results [[1](#_ENREF_1), [3](#_ENREF_3), [4](#_ENREF_4)]. In total, 11 cognitive interviews were executed to refine and finalize the questionnaire. The aim was a heterogeneous sample that would include people from different social groups for which higher rates of response error might be expected. The participants were purposely sampled based on age, mother tongue, and educational level, among other characteristics.

**References**

1. Beatty PC, Willis GB: **Research synthesis: the practice of cognitive interviewing**. *Public Opinion Quarterly* 2007, **71**(2):287-311.

2. Nuyts K, Waege H, Loosveldt G, Billiet J: **Het gebruik van cognitieve interview-technieken bij het ontwikkelen en testen van meetinstrumenten voor survey-onderzoek.** . *Tijdschrift voor Sociologie,* 1997, **18**(4):477-500.

3. Collins D: **Pretesting survey instruments: An overview of cognitive methods'**. *Quality of Life Research* 2003, **12**:229-238.

4. Jobe J, Mingay DJ: **Cognition and survey measurement: History and overview**. *Applied Cognitive Psychology* 1991, **5**:175-193.
