# Supplementary material for: Study protocol: SWING – social capital and well-being in neighborhoods in Ghent
Source: Int J Equity Health. 2015 Apr 9;14:36. doi: 10.1186/s12939-015-0163-1 (PMC4437247; doi:10.1186/s12939-015-0163-1)
Supplement: Additional file 6: — Data processing. [file 12939_2015_163_MOESM6_ESM.docx]

**Additional file 6: Data processing**

***Data handling***

The fieldwork for this study was conducted by trained interviewers, who collected the data and entered them into electronic spread sheets. Strong quality control was used to validate the collected data before entering them in the final database. For all participants, the birth year was known to the researchers (available from the population registry) but not communicated to the interviewers. After survey completion the birth date available from the registry was compared to the self-reported birth year in the survey. When there was a difference between these two variables, the original questionnaire and the data in the spread sheet provided by the interviewer responsible were double-checked. When both variables remained different, the data on this respondent was omitted from the database. Additionally, the original questionnaires were double-checked against the spread sheets to catch any coding errors in the latter. Finally, outliers and impossible data values were identified and cleaned up.

No names or addresses are included in the final database and retracting the identity of the respondents in the database is not possible.

***Data construction***

All measured scale constructs are summative scales of several items; most can be regarded as Likert-type scales. Theoretical considerations as well as factor analyses (forced one-factor solutions in an exploratory principal axis factoring analysis) and reliability analyses were used to construct the final indices. Detailed information on the factor loadings and alpha values can be obtained on request. Although the item non-response was extremely low (not more than 1% for the majority of items) due to the personal interviewing in small groups, we nevertheless decided to use imputation techniques to minimize loss of information in constructing the multiple-item scales and multivariate analyses. The statistical method of Expectation-Maximization (EM) imputation was employed to optimize replacements. This imputation technique was only applied when at least half of the questions within a scale construct were answered. Factor and reliability analyses are based on the imputed variables (results using non-imputed scores are virtually identical).
